# Rituximab vs Cyclophosphamide Induction Therapy for Patients With Granulomatosis With Polyangiitis

**DOI:** 10.1001/jamanetworkopen.2022.43799

**Published:** 2022-11-28

**Authors:** Xavier Puéchal, Michele Iudici, Elodie Perrodeau, Bernard Bonnotte, François Lifermann, Thomas Le Gallou, Alexandre Karras, Claire Blanchard-Delaunay, Thomas Quéméneur, Achille Aouba, Olivier Aumaître, Vincent Cottin, Mohamed Hamidou, Marc Ruivard, Pascal Cohen, Luc Mouthon, Loïc Guillevin, Philippe Ravaud, Raphaël Porcher, Benjamin Terrier

**Affiliations:** 1 National Referral Center for Rare Systemic Autoimmune Diseases, Hôpital Cochin, Assistance Publique–Hôpitaux de Paris (AP-HP) Centre, Université Paris Cité, Paris, France; 2 Division of Rheumatology, Department of Internal Medicine Specialties, Geneva University Hospitals, Geneva, Switzerland; 3 Hôtel-Dieu, AP-HP, Université Paris Cité, Paris, France; 4 Department of Internal Medicine and Clinical Immunology, François Mitterrand University Hospital, Dijon, France; 5 Department of Internal Medicine, Centre Hospitalier Côte-d’Argent, Dax, France; 6 Department of Internal Medicine and Clinical Immunology, Rennes-Sud University Hospital, Rennes, France; 7 Department of Nephrology, Hôpital Européen Georges-Pompidou, AP-HP Centre, Université Paris Cité, Paris, France; 8 Department of Internal Medicine, Centre Hospitalier, Niort, France; 9 Department of Nephrology and Internal Medicine, Centre Hospitalier, Valenciennes, France; 10 Department of Internal Medicine, Côte-de-Nacre University Hospital, Caen, France; 11 Department of Internal Medicine, Gabriel Montpied University Hospital, Clermont-Ferrand, France; 12 National Referral Center for Rare Pulmonary Diseases, Louis-Pradel Hospital, Claude-Bernard University Lyon 1, Lyon, France; 13 Department of Internal Medicine, Hôtel-Dieu University Hospital, Nantes, France

## Abstract

**Importance:**

Results of randomized clinical trials have demonstrated rituximab’s noninferiority to cyclophosphamide as induction therapy for antineutrophil cytoplasm antibody (ANCA)–associated vasculitides (AAV), with neither treatment having a specific advantage for granulomatosis with polyangiitis (GPA). However, post hoc analysis results have suggested that rituximab might be more effective than cyclophosphamide in inducing remission in patients with proteinase 3–positive AAV.

**Objective:**

To compare the effectiveness of rituximab and cyclophosphamide in inducing GPA remission in a large population of unselected patients.

**Design, Setting, and Participants:**

This comparative effectiveness study used multicenter target trial emulation observational data from 32 French hospitals in the French Vasculitis Study Group Registry. Groups were determined according to treatments received, without any intervention from the investigators. Inverse probability of treatment weighting was used to correct for baseline imbalance between groups. Participants included patients with newly diagnosed or relapsing GPA who satisfied American College of Rheumatology classification criteria and/or Chapel Hill Consensus Conference nomenclature. Data were analyzed from October 1, 2021, to May 31, 2022.

**Exposures:**

At least 1 infusion of rituximab or cyclophosphamide for induction therapy between April 1, 2008, and April 1, 2018.

**Main Outcomes and Measures:**

The primary outcome was remission rate at month 6 (±2 months), with remission defined as a Birmingham Vasculitis Activity Score (BVAS) of 0 and prednisone dose of 10 mg/d or less. The BVAS is a validated tool for small-vessel vasculitis and used to assess the level of disease activity, with a numerical weight attached to each involved organ system. The BVAS has a range of 0 to 63 points; a score of 0 indicates no disease activity. Subgroup analyses included the primary outcome for patients with a new diagnosis, for most recently treated patients, and for patients with myeloperoxidase-ANCA positivity.

**Results:**

Among 194 patients with GPA included in the analysis (mean [SD] age, 54 [15] years; 110 men [56.7%]), 165 (85.1%) had a new diagnosis, and 147 of 182 with data available (80.8%) had proteinase 3–ANCA positivity. Sixty-one patients received rituximab and 133 received cyclophosphamide for induction therapy. In the weighted analysis, the primary outcome was reached for 73.1% of patients receiving rituximab vs 40.1% receiving cyclophosphamide (relative risk [RR], 1.82 [95% CI, 1.22-2.73]; risk difference, 33.0% [95% CI, 12.2%-53.8%]; *E* value for RR, 3.05). Similar results were observed in the subgroup of patients with newly diagnosed GPA and those with a more recent treatment. In the subset of 27 patients with myeloperoxidase-ANCA–positive GPA, 8 of 10 rituximab recipients and 8 of 17 cyclophosphamide recipients met the primary end point (unweighted RR, 1.73 [95% CI, 0.96-3.11]).

**Conclusions and Relevance:**

In this comparativeness effectiveness study using clinical data, rituximab induction therapy for GPA was more frequently associated with remission than cyclophosphamide. These results inform clinical decision-making concerning the choice of remission induction therapy for this subset of patients with AAV.

## Key Points

**Question:**

Is rituximab more effective than cyclophosphamide for treatment of granulomatosis with polyangiitis (GPA)?

**Findings:**

In this comparativeness effectiveness study based on clinical data from 194 patients with GPA, rituximab as induction therapy was associated with significantly more achievement of remission with a prednisone dose of 10 mg/d or less compared with cyclophosphamide. Rare disease registries and comparative effectiveness studies enable hitherto poorly addressed treatment comparisons for such entities.

**Meaning:**

These findings may inform clinical decision-making regarding the choice of remission-inducing regimen for patients with GPA.

## Teaser

This comparative effectiveness study compares the association of rituximab vs cyclophosphamide with inducing remission among unselected patients with granulomatosis with polyangiitis.

## Introduction

Antineutrophil cytoplasm antibody (ANCA)–associated vasculitides (AAVs) are potentially life-threatening and commonly relapsing diseases in which necrotizing vasculitis predominantly affects small-to-medium vessels. Among AAVs, granulomatosis with polyangiitis (GPA) is characterized by granulomatous inflammation, usually involving the upper and lower respiratory tract, with frequent necrotizing glomerulonephritis, and with ANCA-targeting proteinase 3 (PR3) in most patients with active disease.^1^ Remission induction treatment of new-onset organ-threatening or life-threatening AAV usually combines glucocorticoids with cyclophosphamide or rituximab.

The diagnosis of GPA as opposed to microscopic polyangiitis is associated with a lower probability of achieving complete remission—defined as no evidence of clinical disease activity while not receiving glucocorticoid treatment^2^—and a higher risk of relapse.^3,4^ Similarly, PR3-ANCA positivity as opposed to myeloperoxidase (MPO)-ANCA positivity is independently associated with a higher risk of relapse.^3,4^

Results of randomized clinical trials^5,6^ showed rituximab’s noninferiority to cyclophosphamide for remission induction in severe AAVs, with neither treatment having a specific advantage for GPA in adjusted analyses. However, post hoc analysis of the trial data^7^ showed that rituximab-treated patients with PR3-AAV achieved complete remission at 6 months more frequently than cyclophosphamide-treated patients. Trial limitations included the enrollment of patients with GPA or microscopic polyangiitis, who might need to be studied separately; small sample size owing to the rarity of those AAVs; and highly selected patients with minor comorbidities that precluded generalization. For those reasons, registry data are useful to complement data from randomized clinical trials to investigate the external validity of drugs prescribed in routine practice. Thus, it remains to be established whether rituximab is more effective than cyclophosphamide at specifically achieving GPA remission. The objective of this study was to undertake an emulation target trial using observational data to compare rituximab vs cyclophosphamide efficacies at inducing remission in a large number of unselected patients with GPA.

## Methods

### Ethics and Regulations

This comparative effectiveness study was conducted in compliance with the Declaration of Helsinki and approved by the Cochin University Hospital Ethics Committee. All patients in the French Vasculitis Study Group (FVSG) Registry gave their written informed consent. This study followed the International Society for Pharmacoeconomics and Outcomes Research (ISPOR) reporting guideline for comparative effectiveness research. This observational analysis was designed to emulate a target trial (ie, a hypothetical pragmatic trial that would have answered the causal question of interest) of induction therapy with rituximab vs cyclophosphamide to obtain GPA remission in a clinical setting.

### Registry

This analysis was based on data from patients with GPA enrolled in the FVSG Registry.^8^ This national registry contains patients’ data entered at least annually from more than 60 French centers. For FVSG Registry inclusion, patients’ GPA diagnoses had to meet the 1990 American College of Rheumatology classification criteria^9^ and/or revised Chapel Hill Consensus Conference nomenclature of vasculitides^1^ considering their entire clinical histories.

### Eligibility Criteria

We included patients with newly diagnosed or relapsing GPA, with active disease defined as a Birmingham Vasculitis Activity Score (BVAS [version 3]) of at least 3^10^ who had received either rituximab or cyclophosphamide (with or without glucocorticoids) for remission induction between April 1, 2008, and April 1, 2018. The BVAS is a validated tool for small-vessel vasculitis and used to assess the level of disease activity, with a numerical weight attached to each involved organ system. The BVAS has a range of 0 to 63 points; a score of 0 indicates no disease activity. Only patients with clinical findings available since diagnosis and who were followed up for at least 6 months or died within 6 months of entry were included. Exclusion criteria were the coexistence of an associated autoimmune disease, active malignant disease or a history of malignant disease in the last 5 years, previous receipt of rituximab for induction therapy, receipt of oral or intravenous cyclophosphamide or rituximab within 4 months before enrollment, history of standard therapy–induced adverse events (ie, bone marrow hypoplasia, cyclophosphamide-induced hemorrhagic cystitis, or malignant disease), limited GPA, alveolar hemorrhage with respiratory failure, creatinine level of greater than 4.0 mg/dL (to convert to μmol/L, multiply by 88.4), or concomitant treatment consisting of plasma exchange(s), methotrexate, mycophenolate mofetil, or azathioprine as part of the induction regimen.

### Interventions

We compared 2 induction therapy strategies according to French guidelines^11^ and used in daily practice during the study period: (1) rituximab administration of 4 weekly infusions of 375 mg/m^2^ or 1 g, 2 weeks apart; and (2) cyclophosphamide infusion of 0.6 g/m^2^ on days 1, 15, and 29, then 0.7 g/m^2^ every 21 days. Patients were allocated to the group corresponding to the first induction agent received (rituximab or cyclophosphamide), regardless of subsequent adherence to this regimen. After achieving remission, maintenance treatments allowed for both groups were azathioprine (2 mg/kg/d), methotrexate (0.3 mg/kg/wk), or a low-dose (500 mg) preemptive rituximab infusion every 6 months from months 4 to 6 through month 24 (4-5 infusions within 18 months).

Patients may have received, or not, 1 to 3 pulses of methylprednisolone (≤1000 mg) followed by oral prednisone equivalent to 1 mg/kg/d (maximum, 80 mg/d). The oral prednisone dose had to be tapered so that, by months 6 (±1 month) and 12 (±1 month), all patients who achieved remission without flaring received prednisone, 10 and 5 mg/d, respectively. Within these parameters, the dose of oral prednisone was allowed at the discretion of the investigator.

### Definitions

The following definitions were applied.^2^ Remission was defined as the absence of signs of new or worsening disease activity according to BVAS of 0^10^ and prednisone dose of 10 mg/d or less; relapse, as the reoccurrence or new appearance of disease activity attributable to active vasculitis. Failure was defined as relapse, all-cause death, addition of or shift to rituximab (for the cyclophosphamide group) or cyclophosphamide (for the rituximab group), or treatment discontinuation. Discontinuation was defined as fewer than 6 infusions (except in documented remission) for the cyclophosphamide group and prescription of oral or intravenous cyclophosphamide within 2 to 6 weeks after induction for the rituximab group. The following manifestations were considered granulomatous manifestations: orbital masses, pulmonary masses, pachymeningitis, subglottic stenosis, or involvement of the ear, nose, and throat.^12^

### Outcomes

The primary outcome measure was remission rate at month 6 (±2 months), as previously defined. Subgroup analyses included the primary outcome for patients with newly diagnosed disease, among those most recently treated, and for patients with MPO-ANCA positivity. Secondary end points were the percentage of patients with BVAS of 0 at month 6 (±2 months) at any glucocorticoid dose, retention rate without failure at 24 months, and safety at month 6 (±2 months). Follow-up for each patient started at induction onset (baseline).

### Statistical Analysis

Data were analyzed from October 1, 2021, to May 31, 2022. An inverse probability of treatment weighting (IPTW) approach was used to account for differences in baseline variables between treatment groups.^13^ Accordingly, observations were weighted by 1 / PS for patients receiving rituximab and 1 / (1 – PS) for those receiving cyclophosphamide, where PS denotes the propensity score (ie, the probability of a patient receiving rituximab for induction, given her or his baseline covariates). The propensity score was estimated using logistic regression. Covariates were selected based on a nonparsimonious approach to account for both potential confounders and variables that could serve as proxies for unknown or unmeasured confounders. Moreover, priority was given to prognostic variables, because they allow lowering of the IPTW-estimator variance, whereas variables strongly associated with treatment but not—or weakly—with the outcome (instrumental variables) can induce unstable weights with little or no gain in terms of bias reduction. Covariates included in the model were year of induction treatment, pure granulomatous disease, BVAS, age, self-reported sex, hematuria, PR3-ANCA positivity, ischemic abdominal pain, stroke, mononeuritis multiplex, severe alveolar hemorrhage or acute respiratory distress syndrome, sensorial deafness, motor peripheral neuropathy, scleritis, creatinine clearance of greater than 30 mL/min, and cranial nerve involvement.

To check the ability of IPTW to obtain well-balanced arms, standardized differences between arms before and after weighting were computed. A standardized difference less than 10% indicated successful balance^14,15^ and served as a target in the propensity score construction. Another key requirement for IPTW was the positivity assumption, that is, the propensity score must be bounded away from 0 and 1. Positivity was assessed by determining the mean stabilized weights and their SD, minimum, and maximum. A mean far from 1 or the presence of very extreme values can indicate nonpositivity or misspecification of the propensity score model.^16^ The overlap of propensity score distributions by group was also assessed graphically, including when the analysis was restricted to patients with newly diagnosed GPA.

To preserve the sample size of the original data, stabilized weights were used.^17^ A truncation at the 99th percentile of the propensity score was also applied to avoid extreme weights. The primary outcome analysis relied on the IPTW relative risk (RR), with 95% CI derived from a Poisson model with robust variance. The absolute risk difference and 95% CI were also estimated using a robust Poisson model with identity link. Two sensitivity analyses were computed for the primary outcome. First, estimates were computed in the unweighted sample. Second, a double-robust estimator for the RR was used. That latter approach combines 2 models: the propensity score model for the IPTW and a Poisson model relating the outcome to the treatment and covariates. This allows an unbiased RR estimate if either the Poisson models for the outcome or the propensity score model is correctly specified, but not necessarily both (double-robustness property). We also evaluated how sensitive the results were to unmeasured confounding using the *E* value, which measures the minimum strength of association that an unmeasured confounder should have with the treatment and outcome to fully exclude the treatment effect.^18^

A preplanned subgroup analysis examined the primary outcome for the subset of patients with newly diagnosed GPA, for those most recently treated, or for those positive for MPO-ANCA. Because the sample size was too small to calculate relevant weights for the latter, we calculated only the unweighted RR (on imputed data sets) based on a Poisson model. The rate of patients with BVAS of 0 at month 6 (±2 months) at any glucocorticoid dose was analyzed using the same approach as the primary outcome. As an exploratory analysis, the hazard ratio with 95% CI for survival without failure at 24 months was estimated in the IPTW sample using a Cox proportional hazards model with robust variance.

Missing baseline variables and outcomes were handled by multiple imputation by chained equation, using all baseline propensity score model variables and the treatment received in the imputation model. The number of independent imputed data sets was determined by rounding the percentage of observations with missing data up to the nearest integer (n = 42). For each data set, a propensity score was estimated, and the linear predictive factors were then pooled according to Rubin rules.^19^ All tests applied a 2-sided *P* ≤ .05 significance level. The analyses were computed using R software, version 4.0.3 (R Foundation for Statistical Computing).

## Results

### Study Population

Among 281 patients with GPA assessed for eligibility, 194 (mean [SD] age, 54 [15] years; 110 men [56.7%] and 84 women [43.3%]) were included (Figure); 61 received rituximab and 133 received cyclophosphamide as induction therapy. Among these, GPA was newly diagnosed in 165 (85.1%); 147 of 182 patients with data available (80.8%) had positive findings for PR3-ANCA. Patients’ main baseline characteristics are reported in Table 1. The median dates of diagnosis were July 2013 for the rituximab group and November 2010 for the cyclophosphamide group.

**Figure.**
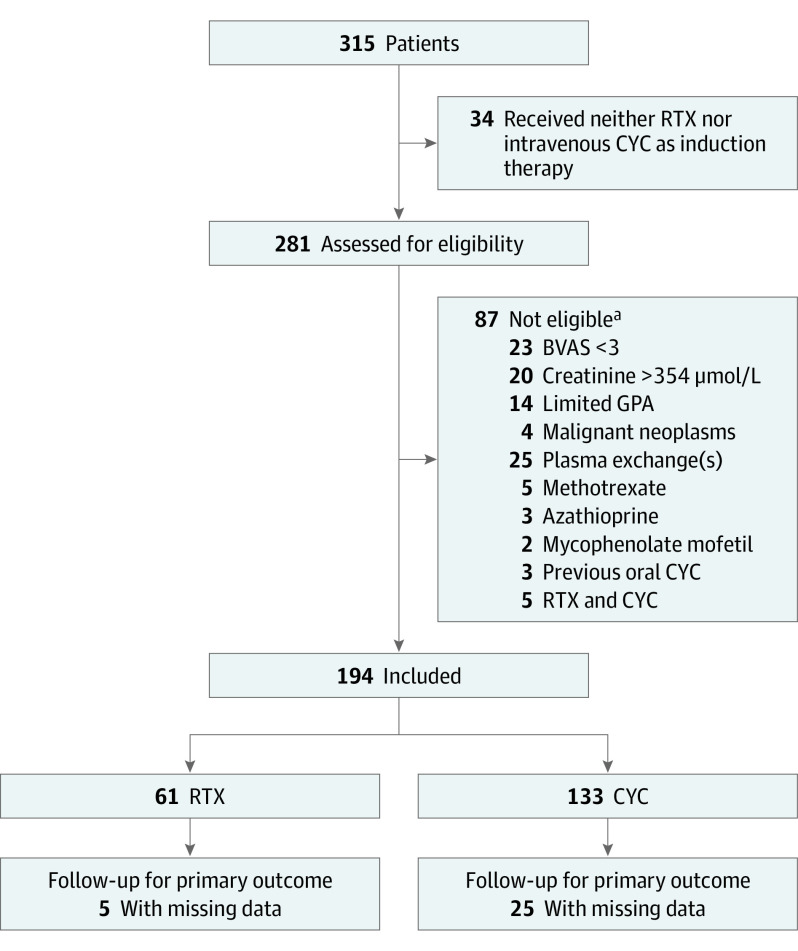
Flowchart of Patients With Granulomatosis With Polyangiitis (GPA) Who Received Rituximab (RTX) or Cyclophosphamide (CYC) as Induction Therapy BVAS indicates Birmingham Vasculitis Activity Score. The BVAS is a validated tool for small-vessel vasculitis and used to assess the level of disease activity, with a numerical weight attached to each involved organ system. The BVAS has a range of 0 to 63 points; a score of 0 indicates no disease activity. ^a^Patients could have more than 1 ineligibility criteria.

**Table 1.** Patient Characteristics at Baseline^a^

Characteristic	All (N = 194)	Rituximab group (n = 61)	Cyclophosphamide group (n = 133)
Age, mean (SD), y	54 (15)	52 (18)	55 (14)
Sex			
Male	110 (56.7)	37 (60.7)	73 (54.9)
Female	84 (43.3)	24 (39.3)	60 (45.1)
New diagnosis	165 (85.1)	34 (55.7)	131 (98.5)
Relapsing disease	29 (14.9)	27 (44.3)	2 (1.5)
PR3-ANCA positive^b^	147 (80.8)	46 (78.0)	101 (82.1)
MPO-ANCA positive^c^	29 (17.5)	10 (17.5)	19 (17.4)
BVAS, mean (SD)^d^	15 (8)	14 (8)	16 (8)
Pure granulomatous disease^e^	84 (48.8)	13 (33.3)	71 (53.4)
Neurological involvement^f^			
Any	57 (34.5)	10 (31.3)	47 (35.3)
Pachymeningitis^f^	2 (1.2)	0	2 (1.5)
Meningitis^f^	3 (1.8)	3 (9.4)	0
Cord lesions^f^	1 (0.6)	1 (3.1)	0
Stroke^f^	5 (3.0)	2 (6.3)	3 (2.3)
Cranial nerve^f^	3 (1.8)	1 (3.1)	2 (1.5)
Mononeuritis multiplex	17 (8.8)	6 (9.8)	11 (8.3)
Motor neuropathy	14 (7.2)	4 (6.6)	10 (7.5)
Skin involvement	65 (33.5)	19 (31.1)	46 (34.6)
Ocular involvement			
Any	44 (22.7)	8 (13.1)	36 (27.1)
Scleritis	14 (7.2)	4 (6.6)	10 (7.5)
Ear, nose, and throat involvement			
Any	146 (75.3)	39 (63.9)	107 (80.5)
Sensorial deafness	4 (2.1)	1 (1.6)	3 (2.3)
Lung involvement			
Any	119 (61.3)	28 (45.9)	91 (68.4)
ARDS	1 (0.5)	0	1 (0.8)
Severe alveolar hemorrhage	27 (13.9)	8 (13.1)	19 (14.3)
Vascular involvement^f^	36 (21.8)	11 (34.4)	25 (18.8)
Cardiomyopathy	6 (3.1)	5 (8.2)	1 (0.8)
Digestive involvement	17 (8.8)	3 (4.9)	14 (10.5)
Kidney involvement^g^			
Any	100 (52.9)	24 (42.9)	76 (57.1)
Hematuria	71 (36.6)	20 (32.8)	51 (38.3)
Creatinine clearance, mean (SD), mL/min^h^	75 (34)	84 (37)	71 (32)
Creatinine clearance >60 mL/min^h^	103 (65.6)	32 (72.7)	71 (62.8)

Abbreviations: ANCA, antineutrophil cytoplasm antibodies; ARDS, acute respiratory distress syndrome; BVAS, Birmingham Vasculitis Activity Score (version 3); MPO, myeloperoxidase; PR3, proteinase 3.

^a^ Unless otherwise indicated, data are expressed as No. (%) of patients. Percentages have been rounded and may not total 100.

^b^ Data available for 182 patients: 59 in the rituximab group and 123 in the cyclophosphamide group.

^c^ Data available for 166 patients: 57 in the rituximab group and 109 in the cyclophosphamide group.

^d^ The BVAS is a validated tool for small-vessel vasculitis and used to assess the level of disease activity, with a numerical weight attached to each involved organ system. The BVAS has a range of 0 to 63 points; a score of 0 indicates no disease activity. Persistent scores can range from 0 to 33, whereas the new/worse scores can range from 0 to 63.

^e^ Data available for 172 patients: 39 in the rituximab group and 133 in the cyclophosphamide group.

^f^ Data available for 165 patients: 32 in the rituximab group and 133 in the cyclophosphamide group.

^g^ Data available for 189 patients: 56 in the rituximab group and 133 in the cyclophosphamide group.

^h^ Data available for 157 patients: 44 in the rituximab group and 113 in the cyclophosphamide group.

### Primary Outcome Analysis

According to the unadjusted analysis, a higher percentage of rituximab recipients (40 of 54 [74.1%]) than cyclophosphamide-treated patients (42 of 107 [39.3%]) achieved BVAS of  0 at 6 months (±2 months) with a prednisone dose of 10 mg/d or less (primary outcome). The weighted analysis on the multiply imputed data sets found that the primary outcome was reached for 73.1% of rituximab recipients vs 40.1% of cyclophosphamide recipients (RR, 1.82 [95% CI, 1.22-2.73]; *E* value for RR, 3.05) (Table 2). The results were similar when the analysis was restricted to those with newly diagnosed GPA or those most recently treated. The subgroup analysis of 27 patients with MPO-ANCA–positive GPA showed that 8 of 10 rituximab recipients and 8 of 17 cyclophosphamide recipients met the primary end point (unweighted RR, 1.73 [95% CI, 0.96-3.11]). To explore a potential association of study center with treatment efficacy, we conducted a sensitivity analysis where the primary outcome was reassessed in a sample excluding 1 center at a time. We found no evidence of such an association (eFigure 1 in Supplement 1).

**Table 2.** Comparative Analyses for Rate of Remission and Achievement of BVAS of 0 at 6 (±2) Months^a^

Outcome	Analysis	Treatment group, %^b^		Relative risk (95% CI)	Difference, % (95% CI)	*E* value
		Rituximab (n = 61)	Cyclophosphamide (n = 133)			
Primary: Comparative analyses for remission rate at 6 (±2) mo	Primary	73.1	40.1	1.82 (1.22 to 2.73)	33.0 (12.2 to 53.8)	3.05
	Unweighted	72.5	40.8	1.78 (1.34 to 2.35)	31.7 (16.5 to 46.8)	2.95
	Double robust	78.4	40.4	1.93 (1.31 to 2.84)	37.9 (14.4 to 61.5)	3.26
	Adjusted^c^	NA	NA	1.90 (1.28 to 2.81)	35.1 (14.5 to 55.6)	3.20
Subgroup analyses restricted to patients with newly diagnosed GPA	Primary	76.1	41.6	1.83 (1.34 to 2.50)	34.5 (15.7 to 53.3)	3.06
	Unweighted	71.1	41.4	1.72 (1.24 to 2.38)	29.7 (10.5 to 48.8)	2.83
	Double robust	81.4	41.1	1.98 (1.48 to 2.65)	40.3 (22.6 to 58.1)	3.37
	Adjusted^d^	NA	NA	1.74 (1.14 to 2.68)	32.2 (8.1 to 56.2)	2.88
Subgroup analyses restricted to patients with most recently treated GPA	Primary	75.2	44.5	1.69 (1.19 to 2.40)	30.7 (12.3 to 49.1)	2.77
	Unweighted	73.2	43.3	1.69 (1.21 to 2.38)	30.0 (12.5 to 47.5)	2.78
	Double robust	76.1	43.2	1.77 (1.29 to 2.42)	33.0 (16.9 to 49.1)	2.93
	Adjusted^e^	NA	NA	1.80 (1.26 to 2.56)	34.4 (15.7 to 53.1)	3.00
Secondary: Comparative analyses for BVAS of 0 achievement at 6 (±2) mo^f^	Primary	85.5	83.2	1.03 (0.85 to 1.24)	2.3 (–13.6 to 18.2)	1.19
	Unweighted	84.9	80.8	1.05 (0.91 to 1.21)	4.1 (–8.0 to 16.1)	1.28

Abbreviations: BVAS, Birmingham Vasculitis Activity Score (version 3); GPA, granulomatosis with polyangiitis; NA, not applicable.

^a^ Analyses are imputed and weighted unless stated otherwise.

^b^ The numbers of patients are weighted and imputed and are therefore not listed.

^c^ Covariates in the adjusted analysis are year of induction, proteinase 3–antineutrophil cytoplasm antibodies positivity, and severe alveolar hemorrhage or acute respiratory distress syndrome.

^d^ Covariates in the adjusted analysis are year of induction, pure granulomatous disease, and proteinase 3–antineutrophil cytoplasm antibodies positivity.

^e^ Covariate in the adjusted analysis is year of induction.

^f^ The BVAS is a validated tool for small-vessel vasculitis and used to assess the level of disease activity, with a numerical weight attached to each involved organ system. The BVAS has a range of 0 to 63 points; a score of 0 indicates no disease activity.

The overlap of propensity score distributions by group is shown graphically (eFigure 2 in Supplement 1), with the distribution of the truncated weights and the standardized differences of main baseline variables before and after IPTW (eTable 1 and eFigure 3 in Supplement 1). The same analyses are shown when restricted to patients with newly diagnosed GPA (eFigures 4 and 5 and eTable 2 in Supplement 1) or those most recently treated (eFigures 6 and 7 and eTable 3 in Supplement 1).

### Secondary Outcome Analysis

For the rituximab group, the number of patients who achieved BVAS of 0 at 6 (±2 months), regardless of prednisone dose, was 47 of 55 (85.5%); in the cyclophosphamide group, 95 of 115 (82.6%). The comparative imputed analyses are shown in Table 2.

Among rituximab induction recipients, rituximab was prescribed as maintenance therapy for 48 of 55 (87.3%); azathioprine, for 2 of 47 (4.3%); methotrexate, for 1 of 48 (2.1%); and mycophenolate mofetil, for 1 of 48 (2.1%). Among cyclophosphamide induction recipients, rituximab was prescribed as maintenance therapy for 57 of 121 (47.1%); azathioprine, for 57 of 117 (48.7%); methotrexate, for 8 of 110 (7.3%); and mycophenolate mofetil, for 7 of 116 (6.0%). Postinclusion treatment failures at 24 months occurred in 7 rituximab recipients and 51 cyclophosphamide recipients, with most due to relapses (in 7 and 33 patients, respectively). Descriptive analysis of treatment failures at 24 months and comparative analyses of survival without failure at 24 months are reported in eTables 4 and 5, respectively, in Supplement 1. Kaplan-Meier estimations of failure rates are shown in eFigure 8 in Supplement 1. No increased toxicity signal was observed for rituximab compared with cyclophosphamide recipients (Table 3).

**Table 3.** Adverse Events at 6 (±2) Months^a^

Adverse event	All (n = 194)	Rituximab group (n = 61)	Cyclophosphamide group (n = 133)
≥1 Severe	8 (4.1)	1 (1.6)	7 (5.2)
Death	1 (0.5)	0	1 (0.8)
Opportunistic infection	0	0	0
Malignant disease	1 (0.5)	0	1 (0.8)
Cardiovascular	1 (0.5)	0	1 (0.81)
Other severe	6 (3.1)	1 (1.6)	5 (3.8)

^a^ Data are presented as the No. (%) of patients.

## Discussion

The results of this target trial emulation based on clinical data of patients with newly diagnosed or relapsing GPA showed that those who received rituximab for induction treatment more frequently achieved remission with a prednisone dose of 10 mg/d or less compared with cyclophosphamide-treated patients. This comparative effectiveness analysis using observational data from a prospective rare disease registry allowed treatment comparisons—so far poorly addressed—and confirmed the hypothesis that rituximab could be superior to cyclophosphamide for induction of GPA remission.

Our findings are consistent with those of the pivotal trial on rituximab induction treatment for AAVs.^6^ In that study, patients with AAV allocated to receive rituximab experienced proportionally (but not significantly) more frequent remissions with successful completion of prednisone tapering at 6 months (64%), compared with patients allocated to receive cyclophosphamide (53%). Their sensitivity analysis restricted to patients with GPA, in line with our observations, highlighted that more rituximab recipients (63% vs 50% of cyclophosphamide recipients) achieved complete remission with successful completion of prednisone tapering at 6 months, even in the absence of a statistically significant difference. Furthermore, a post hoc analysis of trial data from 131 patients with PR3-AAV (127 of whom were classified as having GPA)^7^ showed that patients with PR3-AAV were more than twice as likely to achieve complete remission at 6 months when treated with rituximab rather than with cyclophosphamide (65% vs 48%, respectively; *P* = .04). Differences in key AAV outcomes may not be apparent in clinical trials owing to their own limitations, including the fact that more than 1 disease had often been included to overcome their rarity, and, hence, they were not powered to detect significant differences about a given intervention’s efficacy within a single disease entity.^20^ Our clinical data from a larger subset of patient with GPA indicated a specific advantage of rituximab over cyclophosphamide induction therapy for patients classified as having GPA, most of whom had PR3-ANCA–positive disease.

Our small number of patients with relapsing GPA prescribed cyclophosphamide induction treatment post relapse may be attributed to the known lower cyclophosphamide efficacy to induce AAV remission in such patients found in the pivotal trial.^6^ The higher remission rate among rituximab recipients was not owing to more of them having relapsing disease. Our analysis confirmed that remission rate remained significantly higher for rituximab recipients than cyclophosphamide recipients when the analysis was restricted to those with newly diagnosed GPA, a finding not yet reported.

Between-group differences in the numbers of patients achieving 6-month BVAS of 0 at any glucocorticoid dose was not observed, which might be attributed to residual vasculitis activity that was undetectable because of glucocorticoid masking. It may be easier to demonstrate treatment efficacy by including the glucocorticoid dose in the definition of the primary end point, as recommended,^21^ rather than using the BVAS alone. The absence of differences for patients with MPO-GPA may only reflect the small sample size. Although secondary outcome analysis results also gave the advantage to rituximab over cyclophosphamide, the data are unclear because more recipients of rituximab induction treatment also received rituximab maintenance therapy, known to be more effective. No toxicity signals were observed in either group.

### Strengths and Limitations

Our study has major strengths. The FVSG Registry collected the clinical characteristics and outcomes of 194 patients originating from several medical specialties, thereby enabling analysis of a truly representative distribution of different GPA presentations and evolutions. The thoroughness of these data allowed detailed characterizations of both treatment groups, with well-phenotyped baseline features and outcome ascertainment and close matching according to key confounders. Analyses benefited from multiple methods to address potential confounding by indication, and subgroup and sensitivity analyses were concordant for the primary outcome, bolstering confidence in the main results. The demographic composition of the FVSG Registry allowed inclusion of patients with comorbidities and/or with MPO-ANCA–positive and ANCA-negative disease, who are often underrepresented.

This study has some limitations. First, as in any observational analysis, assignment to a particular treatment was not randomized. The higher number of rituximab recipients with relapsing disease could not be balanced because of the small number of such cyclophosphamide recipients. This enrichment cannot explain the overall higher remission rate of recipients of rituximab induction treatment, as supported by the analysis restricted to newly diagnosed patients. In addition, we rigorously balanced the rituximab and cyclophosphamide groups with respect to all other demographic characteristics, medical history, and all covariates a priori thought to be associated with the likelihood of receiving rituximab or cyclophosphamide and achieving remission. However, we agree that other unmeasured confounding that could affect the primary outcome might still persist. Second, although the enrolment period was limited to 10 years, it cannot be excluded that the physicians gradually attempted a transition toward lower levels of glucocorticoid use for patients with AAV during the induction phase, which might have benefited rituximab recipients, who had received induction treatment at a later median date than the cyclophosphamide group. However, the year in which patients received their induction treatment was taken into account in the statistical analysis. Third, patients with limited GPA, alveolar hemorrhage with respiratory failure, or creatinine level greater than 4.0 mg/dL (to convert to μmol/L, multiply by 88.4) or who received concomitant treatment with plasma exchange(s), methotrexate, or azathioprine as part of their induction regimen were excluded. The comparative efficacy of rituximab vs cyclophosphamide regimens is less clear in these patients. However, they represent only a minority of our patients who were recruited from 32 vasculitis centers, including several medical specialties. Thus, we think that our data are likely to be representative of the entire GPA spectrum and that our sample is a good representation of patients in whom the question of induction with rituximab or cyclophosphamide arises. Additionally, the secondary outcome analysis results also supported rituximab over cyclophosphamide, but the data remain uncertain, because more rituximab induction recipients also received rituximab for maintenance, and the analyses did not account for treatments given after the exposure of interest.

## Conclusions

The findings of this target trial emulation suggest that patients with GPA obtained remission with a prednisone dose of 10 mg/d or less more frequently when they had received rituximab rather than cyclophosphamide for induction therapy. Our results may inform clinical decision-making concerning the choice of remission induction therapy for this subset of patients with AAV.

## Notes

Supplement 1. **eFigure 1.** Sensitivity Analysis Where the Primary Outcome Was Reassessed in a Sample Excluding 1 Center at a Time **eFigure 2.** Propensity Score (Propensity to Receive RTX) for the RTX and CYC Groups (n = 194) Before (A) and After (B) Inverse Probability of Treatment Weighting **eTable 1.** Distribution of the Truncated Weights (99th Percentile) for the RTX and CYC Groups **eFigure 3.** Standardized Differences of Main Baseline Variables Before and After Inverse Probability of Treatment Weighting (n = 194) **eFigure 4.** Propensity Score (Propensity to Receive RTX) Limited to the Subgroup of Newly Diagnosed Patients for the RTX and CYC Groups (n = 165) Before (A) and After (B) Inverse Probability of Treatment Weighting **eTable 2.** Distribution of the Truncated Weights (99th Percentile) for the RTX and CYC Groups in the Subgroup of Newly Diagnosed Patients **eFigure 5.** Standardized Differences of Main Baseline Variables Before and After Inverse Probability of Treatment Weighting in the Subgroup of Newly Diagnosed Patients (n = 165) **eFigure 6.** Propensity Score (Propensity to Receive RTX) Limited to the Subgroup of Most Recently Treated Patients for the RTX and CYC Groups (n = 124) Before (A) and After (B) Inverse Probability of Treatment Weighting **eTable 3.** Distribution of the Truncated Weights (99th Percentile) for the RTX and CYC Groups in the Subgroup of Most Recently Treated Patients **eFigure 7.** Standardized Differences of Main Baseline Variables Before and After Inverse Probability of Treatment Weighting in the Subgroup of Most Recently Treated Patients (n = 124) **eTable 4.** Descriptive Analysis of Failures at 24 Months **eTable 5.** Comparative Analyses of Survival Rate Without Failure at 24 Months **eFigure 8.** Kaplan-Meier Estimations of Failure Rates

Supplement 2.
Nonauthor Collaborators

Supplement 3.
Data Sharing Statement
