# Supplementary material for: Rituximab vs Cyclophosphamide Induction Therapy for Patients With Granulomatosis With Polyangiitis
Source: JAMA Netw Open. 2022 Nov 28;5(11):e2243799. doi: 10.1001/jamanetworkopen.2022.43799 (PMC9706346; doi:10.1001/jamanetworkopen.2022.43799)
Supplement: Supplement 1. — eFigure 1. Sensitivity Analysis Where the Primary Outcome Was Reassessed in a Sample Excluding 1 Center at a Time eFigure 2. Propensity Score (Propensity to Receive RTX) for the RTX and CYC Groups (n = 194) Before (A) and After (B) Inverse Probability of Treatment Weighting eTable 1. Distribution of the Truncated Weights (99th Percentile) for the RTX and CYC Groups eFigure 3. Standardized Differences of Main Baseline Variables Before and After Inverse Probability of Treatment Weighting (n = 194) eFigure 4. Propensity Score (Propensity to Receive RTX) Limited to the Subgroup of Newly Diagnosed Patients for the RTX and CYC Groups (n = 165) Before (A) and After (B) Inverse Probability of Treatment Weighting eTable 2. Distribution of the Truncated Weights (99th Percentile) for the RTX and CYC Groups in the Subgroup of Newly Diagnosed Patients eFigure 5. Standardized Differences of Main Baseline Variables Before and After Inverse Probability of Treatment Weighting in the Subgroup of Newly Diagnosed Patients (n = 165) eFigure 6. Propensity Score (Propensity to Receive RTX) Limited to the Subgroup of Most Recently Treated Patients for the RTX and CYC Groups (n = 124) Before (A) and After (B) Inverse Probability of Treatment Weighting eTable 3. Distribution of the Truncated Weights (99th Percentile) for the RTX and CYC Groups in the Subgroup of Most Recently Treated Patients eFigure 7. Standardized Differences of Main Baseline Variables Before and After Inverse Probability of Treatment Weighting in the Subgroup of Most Recently Treated Patients (n = 124) eTable 4. Descriptive Analysis of Failures at 24 Months eTable 5. Comparative Analyses of Survival Rate Without Failure at 24 Months eFigure 8. Kaplan-Meier Estimations of Failure Rates [file jamanetwopen-e2243799-s001.pdf]

Supplementary Online Content

Puéchal X, Iudici M, Perrodeau E, et al; French Vasculitis Study Group. Rituximab vs cyclophosphamide induction therapy for patients with granulomatosis with polyangiitis. *JAMA Netw Open*. 2022;5(11):e2243799. doi:10.1001/jamanetworkopen.2022.43799

eFigure 1. Sensitivity Analysis Where the Primary Outcome Was Reassessed in a Sample Excluding 1 Center at a Time
eFigure 2. Propensity Score (Propensity to Receive RTX) for the RTX and CYC Groups (n = 194) Before (A) and After (B) Inverse Probability of Treatment Weighting
eTable 1. Distribution of the Truncated Weights (99th Percentile) for the RTX and CYC Groups
eFigure 3. Standardized Differences of Main Baseline Variables Before and After Inverse Probability of Treatment Weighting (n = 194)
eFigure 4. Propensity Score (Propensity to Receive RTX) Limited to the Subgroup of Newly Diagnosed Patients for the RTX and CYC Groups (n = 165) Before (A) and After (B) Inverse Probability of Treatment Weighting
eTable 2. Distribution of the Truncated Weights (99th Percentile) for the RTX and CYC Groups in the Subgroup of Newly Diagnosed Patients
eFigure 5. Standardized Differences of Main Baseline Variables Before and After Inverse Probability of Treatment Weighting in the Subgroup of Newly Diagnosed Patients (n = 165)
eFigure 6. Propensity Score (Propensity to Receive RTX) Limited to the Subgroup of Most Recently Treated Patients for the RTX and CYC Groups (n = 124) Before (A) and After (B) Inverse Probability of Treatment Weighting
eTable 3. Distribution of the Truncated Weights (99th Percentile) for the RTX and CYC Groups in the Subgroup of Most Recently Treated Patients
eFigure 7. Standardized Differences of Main Baseline Variables Before and After Inverse Probability of Treatment Weighting in the Subgroup of Most Recently Treated Patients (n = 124)
eTable 4. Descriptive Analysis of Failures at 24 Months
eTable 5. Comparative Analyses of Survival Rate Without Failure at 24 Months
eFigure 8. Kaplan-Meier Estimations of Failure Rates

This supplementary material has been provided by the authors to give readers additional information about their work.

© 2022 Puéchal X et al. *JAMA Network Open*.

eFigure 1. Sensitivity Analysis Where the Primary Outcome Was Reassessed in a Sample Excluding 1 Center at a Time

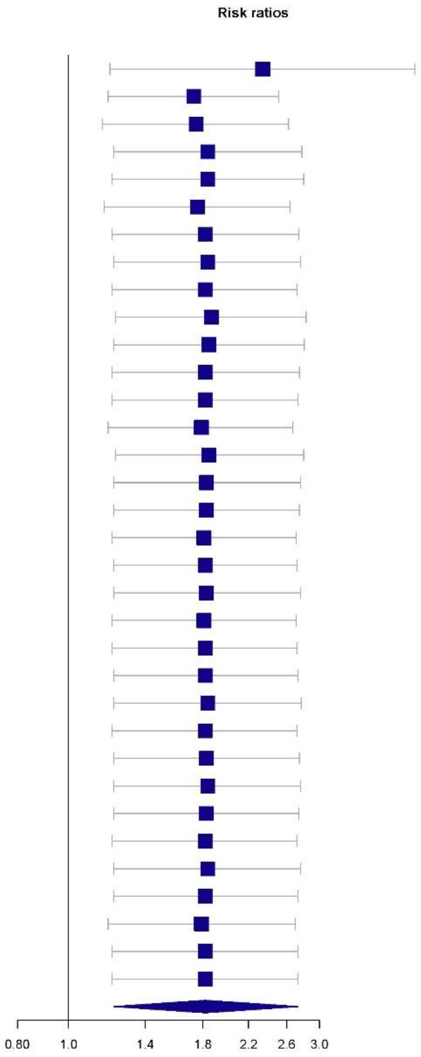

Removed center	N	RTX (%)	CYC (%)	RR (95%CI)
1	89	77.2	32.6	2.34 (1.20 to 4.56)
2	188	73.8	42.8	1.73 (1.19 to 2.51)
3	188	72.6	41.6	1.75 (1.16 to 2.62)
4	188	73.2	39.8	1.84 (1.22 to 2.78)
5	188	72.4	39.3	1.84 (1.21 to 2.80)
6	189	72.6	41.3	1.76 (1.17 to 2.64)
7	189	73.1	40.1	1.82 (1.21 to 2.74)
8	189	74.0	40.3	1.84 (1.22 to 2.76)
9	190	73.4	40.4	1.82 (1.21 to 2.72)
10	190	73.1	39.2	1.87 (1.23 to 2.83)
11	190	73.1	39.4	1.85 (1.22 to 2.81)
12	190	73.1	40.1	1.82 (1.21 to 2.75)
13	191	72.7	40.1	1.82 (1.21 to 2.73)
14	191	73.1	40.9	1.79 (1.19 to 2.67)
15	191	73.1	39.5	1.85 (1.23 to 2.80)
16	191	73.4	40.1	1.83 (1.22 to 2.76)
17	192	73.1	40.0	1.83 (1.22 to 2.75)
18	192	73.1	40.5	1.81 (1.21 to 2.71)
19	192	73.2	40.3	1.82 (1.22 to 2.72)
20	192	73.1	39.9	1.83 (1.22 to 2.76)
21	193	73.1	40.4	1.81 (1.21 to 2.71)
22	193	73.1	40.3	1.82 (1.21 to 2.72)
23	193	73.1	40.1	1.82 (1.22 to 2.73)
24	193	73.1	39.7	1.84 (1.22 to 2.77)
25	193	73.1	40.3	1.82 (1.21 to 2.72)
26	193	73.1	39.9	1.83 (1.22 to 2.75)
27	193	73.1	39.8	1.84 (1.22 to 2.76)
28	193	73.1	40.0	1.83 (1.22 to 2.74)
29	193	73.1	40.3	1.82 (1.21 to 2.72)
30	193	73.1	39.8	1.84 (1.22 to 2.76)
31	193	73.1	40.1	1.82 (1.22 to 2.73)
32	193	71.8	40.1	1.79 (1.19 to 2.70)
33	193	72.9	40.1	1.82 (1.21 to 2.73)
34	193	72.8	40.1	1.82 (1.21 to 2.73)
**All centers**	**194**	**73.1**	**40.1**	**1.82 (1.22 to 2.73)**

© 2022 Puéchal X et al. *JAMA Network Open*.

eFigure 2. Propensity Score (Propensity to Receive RTX) for the RTX and CYC Groups (n = 194) Before (A) and After (B) Inverse Probability of Treatment Weighting

A

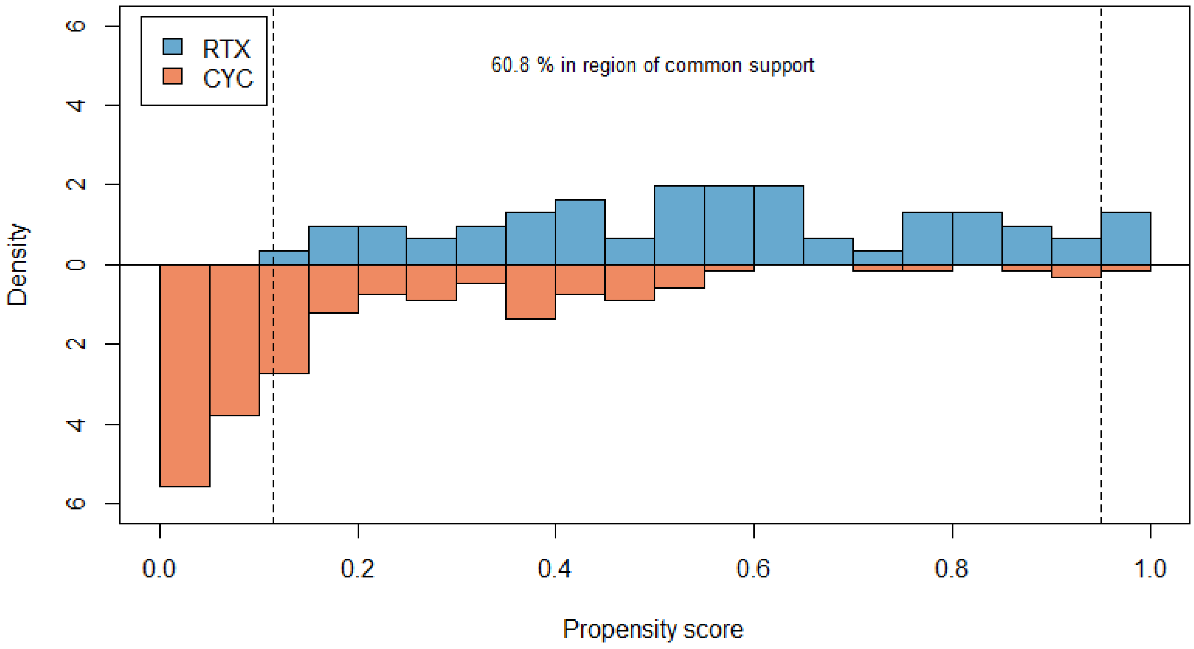

B

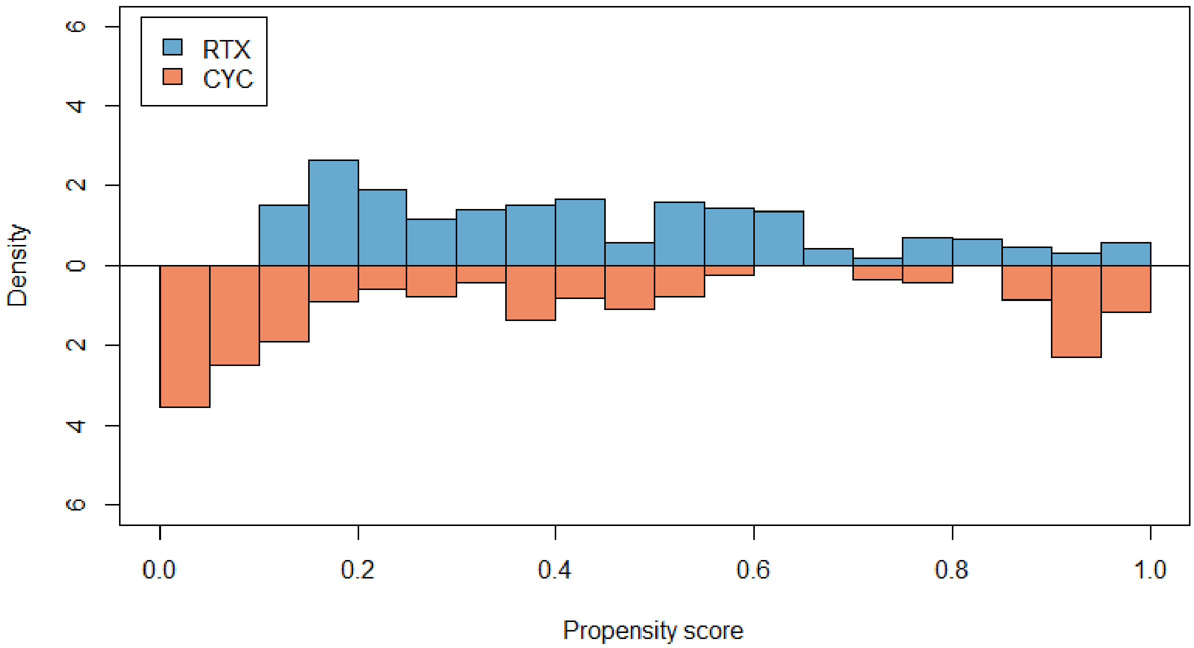

RTX, rituximab; CYC, cyclophosphamide.

© 2022 Puéchal X et al. *JAMA Network Open*.

eTable 1. Distribution of the Truncated Weights (99th Percentile) for the RTX and CYC Groups

	Min.	1st Qu.	Median	Mean	3rd Qu.	Max.	SD
Global	0.32	0.70	0.75	1.00	0.95	8.53	1.11
RTX	0.32	0.41	0.56	0.76	0.82	3.53	0.56
CYC	0.69	0.72	0.77	1.11	1.01	8.53	1.27

© 2022 Puéchal X et al. *JAMA Network Open*.

eFigure 3. Standardized Differences of Main Baseline Variables Before and After Inverse Probability of Treatment Weighting (n = 194)

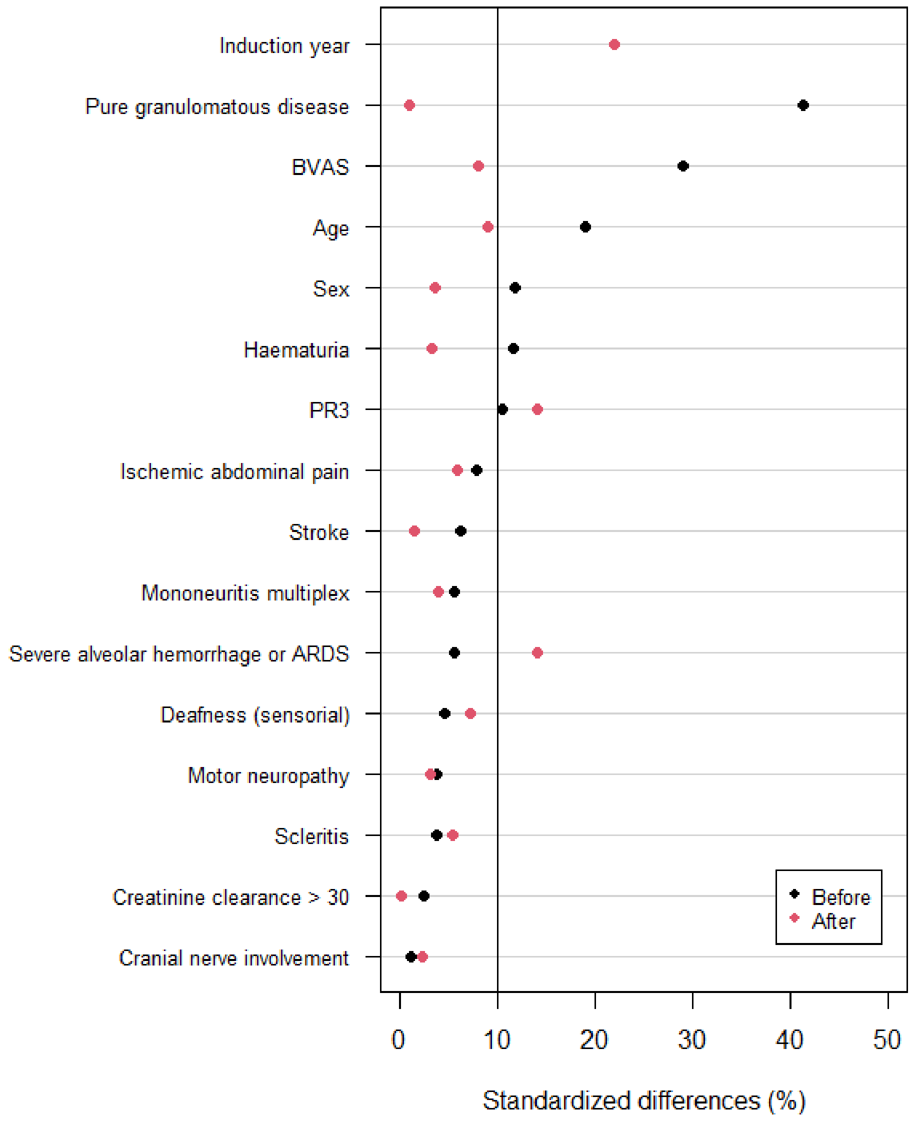

© 2022 Puéchal X et al. *JAMA Network Open*.

eFigure 4. Propensity Score (Propensity to Receive RTX) Limited to the Subgroup of Newly Diagnosed Patients for the RTX and CYC Groups (n = 165) Before (A) and After (B) Inverse Probability of Treatment Weighting

A

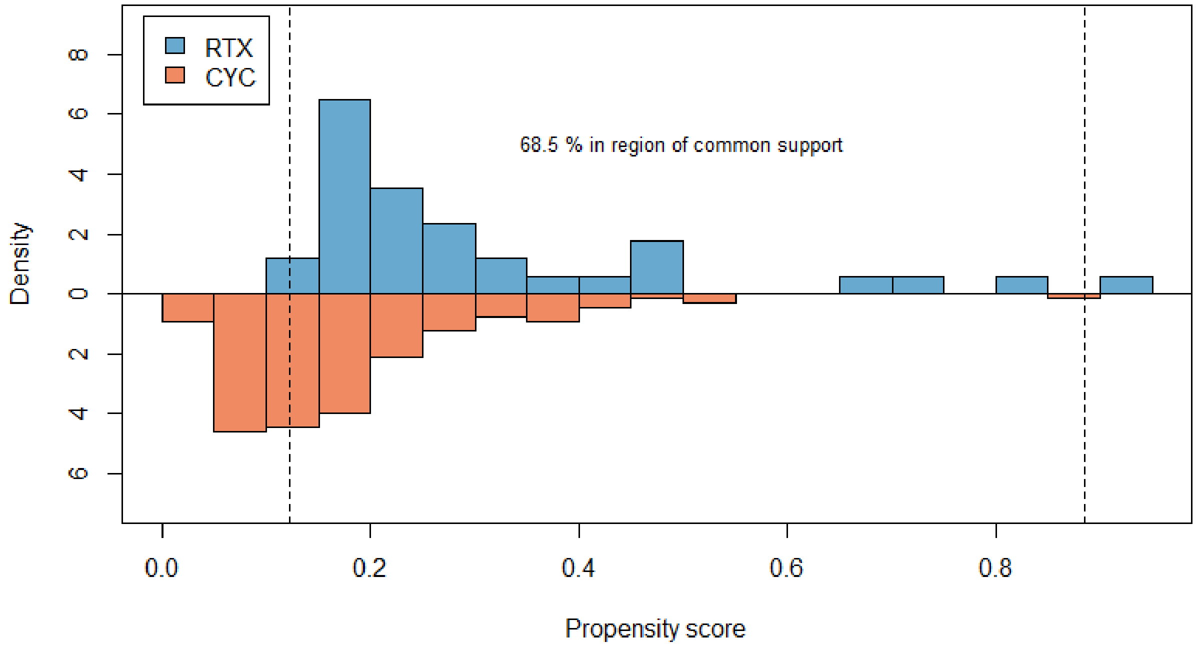

B

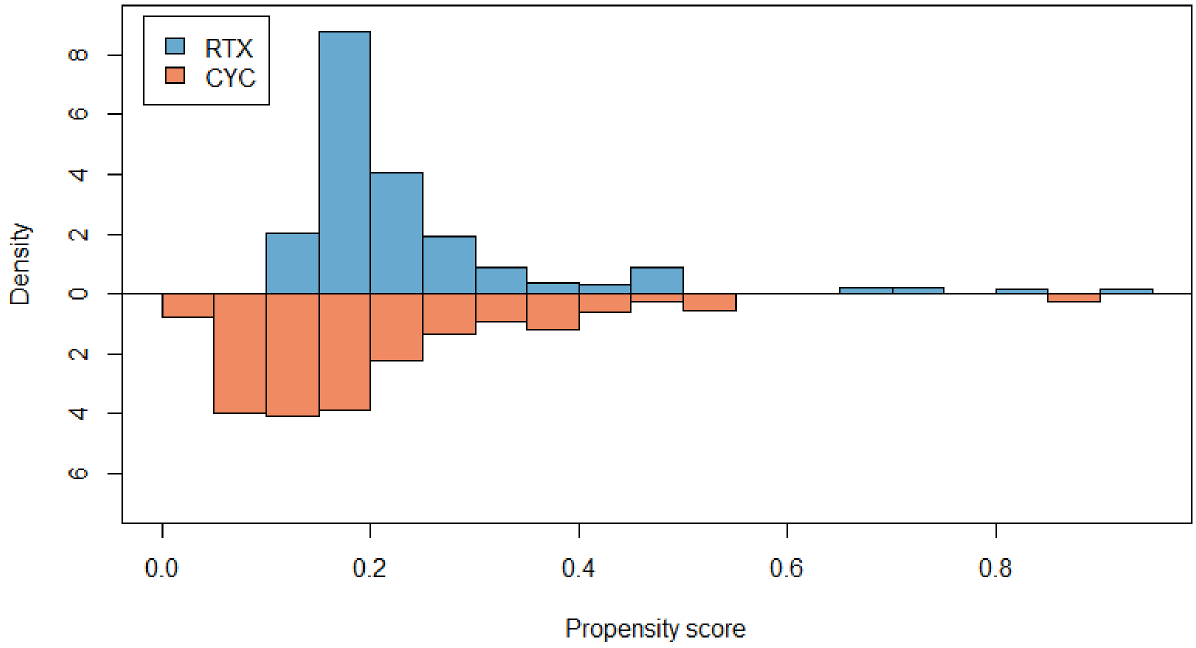

RTX, rituximab; CYC, cyclophosphamide.

© 2022 Puéchal X et al. *JAMA Network Open*.

eTable 2. Distribution of the Truncated Weights (99th Percentile) for the RTX and CYC Groups in the Subgroup of Newly Diagnosed Patients

	Min.	1st Qu.	Median	Mean	3rd Qu.	Max.	SD
Global	0.23	0.86	0.93	0.97	1.05	1.76	0.24
RTX	0.23	0.57	0.87	0.89	1.16	1.71	0.40
CYC	0.82	0.87	0.94	0.99	1.02	1.76	0.18

eFigure 5. Standardized Differences of Main Baseline Variables Before and After Inverse Probability of Treatment Weighting in the Subgroup of Newly Diagnosed Patients (n = 165)

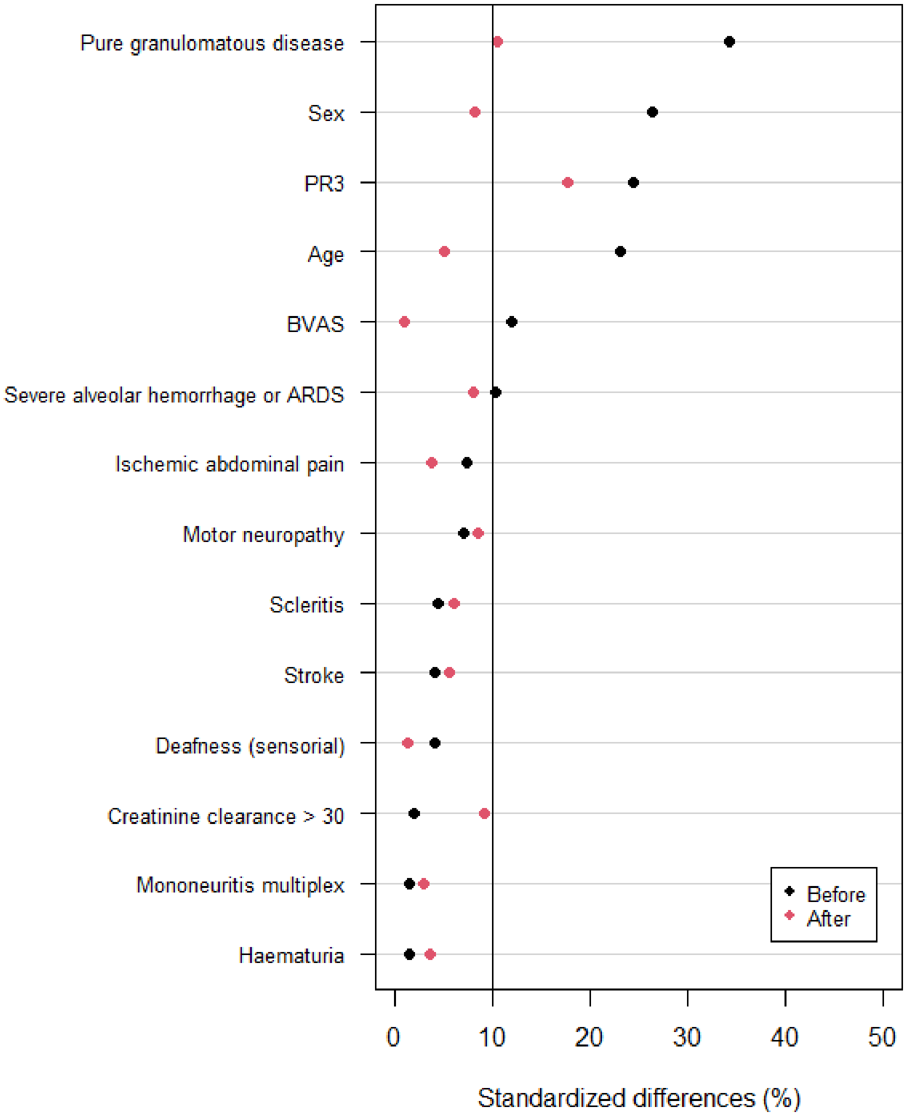

© 2022 Puéchal X et al. *JAMA Network Open*.

eFigure 6. Propensity Score (Propensity to Receive RTX) Limited to the Subgroup of Most Recently Treated Patients for the RTX and CYC Groups (n = 24) Before (A) and After (B) Inverse Probability of Treatment Weighting

A

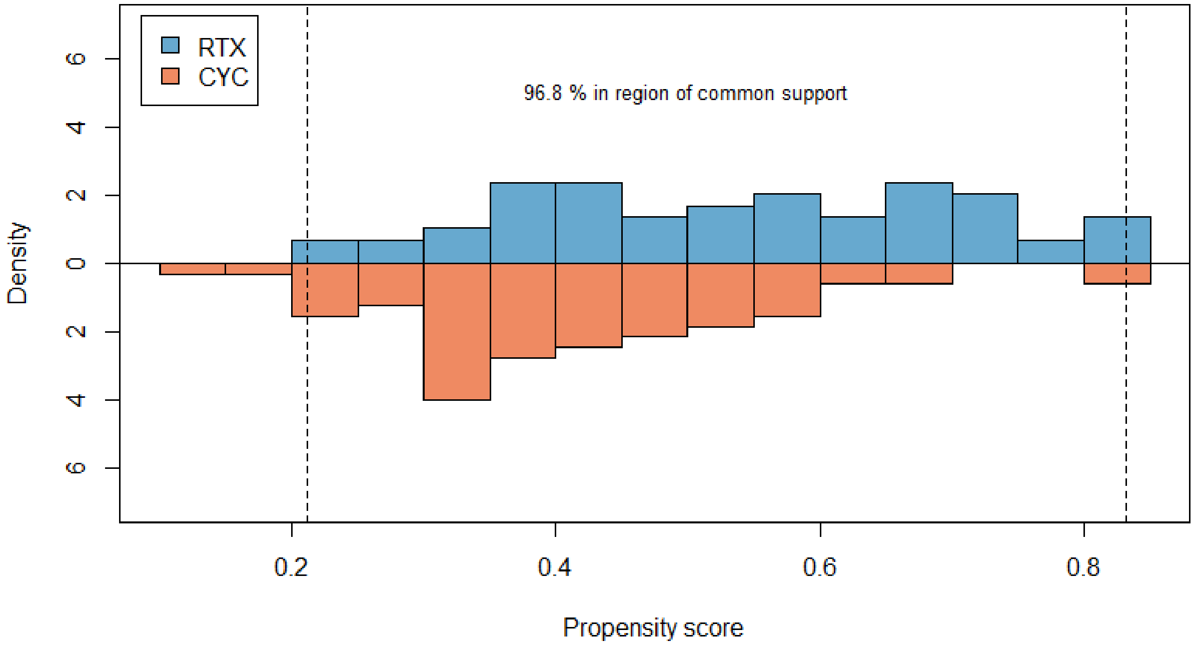

B

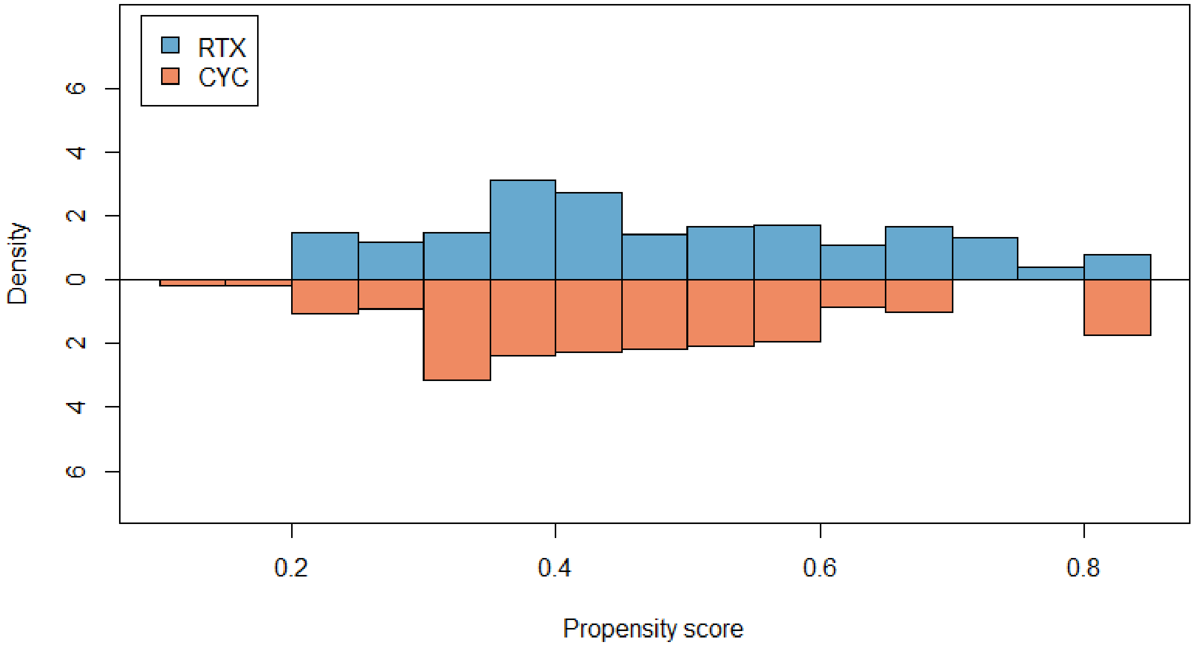

© 2022 Puéchal X et al. *JAMA Network Open*.

eTable 3. Distribution of the Truncated Weights (99th Percentile) for the RTX and CYC Groups in the Subgroup of Most Recently Treated Patients

	Min.	1st Qu.	Median	Mean	3rd Qu.	Max.	SD
Global	0.57	0.76	0.88	1.00	1.14	2.83	0.39
RTX	0.57	0.71	0.95	1.00	1.19	2.33	0.37
CYC	0.59	0.77	0.87	0.99	1.07	2.83	0.40

© 2022 Puéchal X et al. *JAMA Network Open*.

eFigure 7. Standardized Differences of Main Baseline Variables Before and After Inverse Probability of Treatment Weighting in the Subgroup of Most Recently Treated Patients (n = 124)

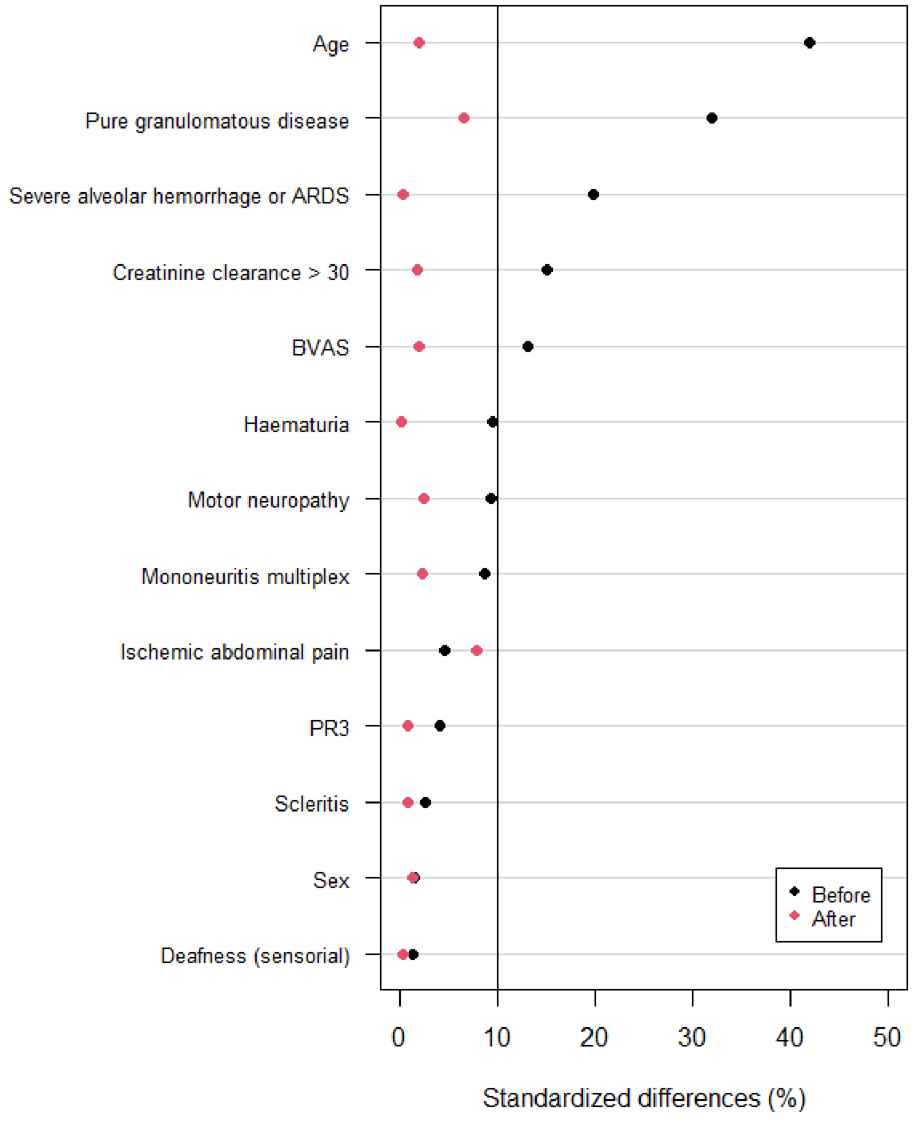

© 2022 Puéchal X et al. *JAMA Network Open*.

eTable 4. Descriptive Analysis of Failures at 24 Months

**Failures**	**RTX**	**CYC**
Total numbers	7	51
Deaths	0	3
Discontinuation	1	10
Relapses	7	33
Add-on	3	26
First-event	7	51
Deaths	0	2
Discontinuation	0	10
Relapses	7	31
Add-on	0	8

51 patients presented 72 failures in the CYC group and 7 patients had 11 failures in the RTX group.
26 patients who received CYC as initial induction therapy were switched to RTX within 24 months, 9 of whom were switched within 6 months. Similarly, 3 patients who had received RTX as initial induction therapy were subsequently switched to CYC, all within 6 months.

eTable 5. Comparative Analyses of Survival Rate Without Failure at 24 Months

**Analysis**	**RTX (n = 61)**	**CYC (n = 133)**	**Hazard ratio [95 CI%]**	**E-value**
Primary	88.3 [79.4; 98.7]	59.7 [51.6; 69.5]	0.24 [0.09; 0.61]	4.74
Unweighted	86.1 [77.1; 96.4]	58.8 [50.9; 68.4]	0.31 [0.14; 0.68]	3.91

© 2022 Puéchal X et al. *JAMA Network Open*.

eFigure 8. Kaplan-Meier Estimations of Failure Rates

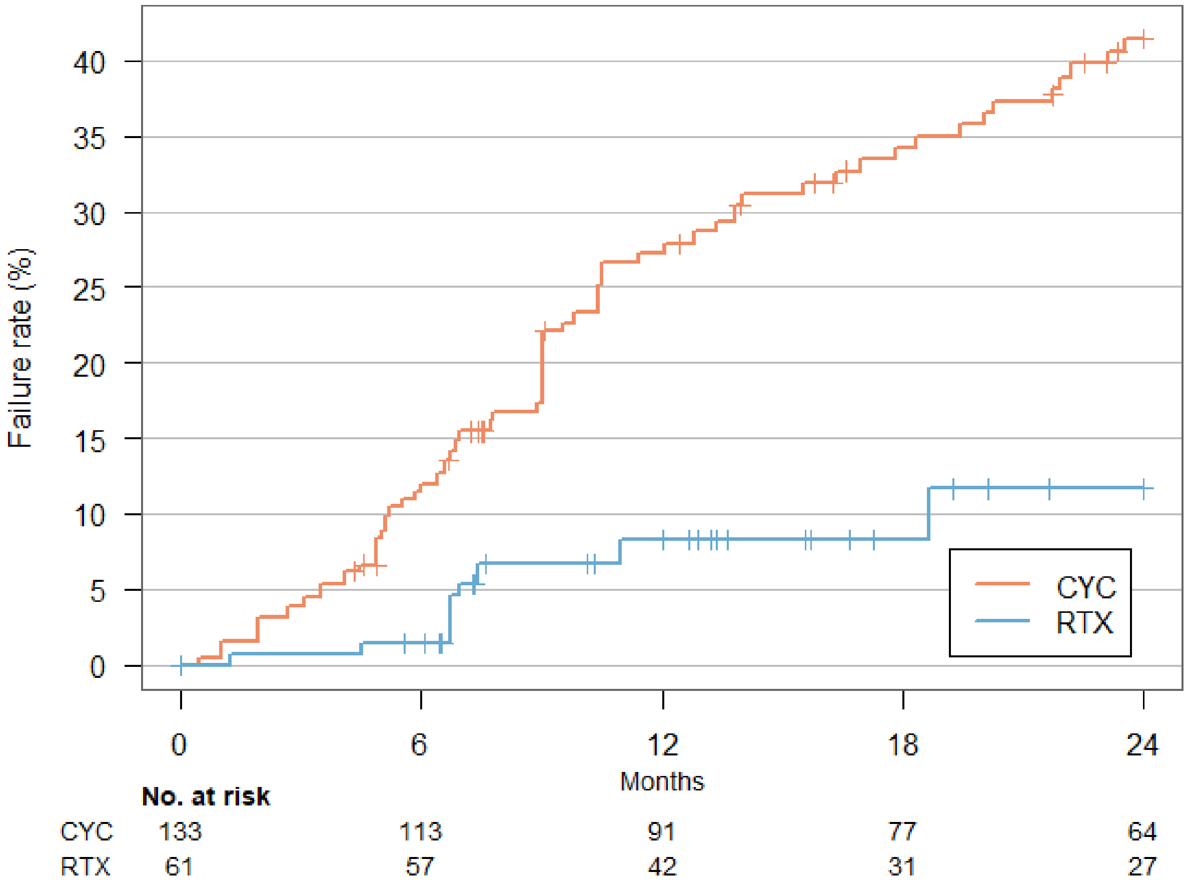

© 2022 Puéchal X et al. *JAMA Network Open*.
