# Supplementary material for: Rituximab vs Cyclophosphamide Induction Therapy for Patients With Granulomatosis With Polyangiitis
Source: JAMA Netw Open. 2022 Nov 28;5(11):e2243799. doi: 10.1001/jamanetworkopen.2022.43799 (PMC9706346; doi:10.1001/jamanetworkopen.2022.43799)
Supplement: Supplement 2. — Nonauthor Collaborators [file jamanetwopen-e2243799-s002.pdf]

Supplemental Online Content: Nonauthor Collaborators
*First name, last name, and suffix (if applicable) are required and will appear in PubMed.

*Group Name(s): French Vasculitis Study Group							
***First Name and Middle Initial(s)**	***Last Name**	***Suffix (eg, Jr, III)**	Academic Degrees	Institution	Location (city, state/province, country)	Role or Contribution, eg, chair, principal investigator	Group (if more than 1 Group listed in the byline) and/or Subgroup (eg, Steering Committee)
Catherine	Achard-Hottelart		MD	department of nephrology, CHU Limoges	Limoges, France	data acquisition	
Badih	Ayach		MD	department of nephrology, CH Charleville-Mézières	Charleville-Mézières, France	data acquisition	
Holy	Bezanahary		MD	department of internal medicine, CHU Dupuytren	Limoges, France	data acquisition	
Jean-Jacques	Boffa		MD, PhD	department of nephrology, CHU Tenon	Paris, France	data acquisition	
Thierry	Colin		MD	department of rheumatology, CH Public du Cotentin	Cherbourg-Octeville, France	data acquisition	
Christophe	Charasse		MD	department of nephrology, CH Saint Brieuc	Saint Brieuc, France	data acquisition	
Isabelle	de Lacroix-Szmania		MD	department of internal medicine, CH Intercommunal	Créteil, France	data acquisition	
Xavier	Delbrel		MD	department of internal medicine, CH Pau	Pau, France	data acquisition	
Hélène	Desmurs-Clavel		MD	department of internal medicine, CHU Edouard Herriot	Lyon, France	data acquisition	
Jean-Jacques	Dion		MD	department of nephrology, CH Charleville-Mézières	Charleville-Mézières, France	data acquisition	
Elisabeth	Diot		MD, PhD	department of internal medicine, CHU Bretonneau	Tours, France	data acquisition	
Bertrand	Godeau		MD, PhD	department of internal medicine, CHU Henri Mondor	Créteil, France	data acquisition	
Guillaume	Gondran		MD	department of internal medicine, CHU Dupuytren	Limoges, France	data acquisition	

Supplemental Online Content: Nonauthor Collaborators
*First name, last name, and suffix (if applicable) are required and will appear in PubMed.

***First Name and Middle Initial(s)**	***Last Name**	***Suffix (eg, Jr, III)**	Academic Degrees	Institution	Location (city, state/province, country)	Role or Contribution, eg, chair, principal investigator	Group (if more than 1 Group listed in the byline) and/or Subgroup (eg, Steering Committee)
Bruno	Graffin		MD	department of internal medicine, Polyclinique Mutualiste	Ollioules, France	data acquisition	
Frédéric	Grassin		MD	department of respiratory medicine, Clinique des Augustines	Malestroit, France	data acquisition	
Catherine	Hanrotel-Saliou		MD	department of nephrology, CHU Cavale Blanche	Brest, France	data acquisition	
Bernard	Imbert		MD	department of internal medicine, CHU Grenoble	Grenoble, France	data acquisition	
Gilles	Jébrak		MD	department of respiratory medicine, CHU Bichat	Paris, France	data acquisition	
Claire	Le Hello		MD, PhD	department of vascular medicine, CHU Saint Etienne	Saint Etienne, France	data acquisition	
Claire	Le Jeunne		MD	department of internal medicine, CHU Cochin	Paris, France	data acquisition	
Bruno	Legallicier		MD	department of internal medicine, CHU Charles Nicolle	Rouen, France	data acquisition	
Nicolas	Limal		MD	department of internal medicine, CHU Henri Mondor	Créteil, France	data acquisition	
Véronique	Loustaud-Ratti		MD	department of internal medicine, CHU de Limoges	Limoges, France	data acquisition	
Isabelle	Marie		MD, PhD	department of internal medicine, CHU Charles Nicolle	Rouen, France	data acquisition	
Dominique	Merrien		MD	department of internal medicine, CH Vendée	La Roche-sur-Yon, France	data acquisition	
Marc	Michel		MD, PhD	department of internal medicine, CHU Henri Mondor	Créteil, France	data acquisition	

Supplemental Online Content: Nonauthor Collaborators
*First name, last name, and suffix (if applicable) are required and will appear in PubMed.

***First Name and Middle Initial(s)**	***Last Name**	***Suffix (eg, Jr, III)**	Academic Degrees	Institution	Location (city, state/province, country)	Role or Contribution, eg, chair, principal investigator	Group (if more than 1 Group listed in the byline) and/or Subgroup (eg, Steering Committee)
Antoine	Néel		MD, PhD	department of internal medicine, CHU Nantes	Nantes, France	data acquisition	
Yann	Olivier		MD	department of internal medicine, CHU Côte de Nacre	Caen, France	data acquisition	
Elisa	Pasqualoni		MD	department of internal medicine, CH Saint Denis	Saint Denis, France	data acquisition	
Alexis	Régent		MD PhD	department of internal medicine, CHU Cochin	Paris, France	data acquisition	
Jérome	Rossert		MD, PhD	department of nephrology, CHU Tenon	Paris, France	data acquisition	
Eric	Thervet		MD, PhD	department of nephrology, Hôpital Européen Georges Pompidou	Paris, France	data acquisition	
Stéphane	Vinzio		MD	department of internal medicine, Groupe Hospitalier Mutualiste	Grenoble, France	data acquisition	
