# Supplementary material for: Rituximab vs Cyclophosphamide Induction Therapy for Patients With Granulomatosis With Polyangiitis
Source: JAMA Netw Open. 2022 Nov 28;5(11):e2243799. doi: 10.1001/jamanetworkopen.2022.43799 (PMC9706346; doi:10.1001/jamanetworkopen.2022.43799)
Supplement: Supplement 3. — Data Sharing Statement [file jamanetwopen-e2243799-s003.pdf]

Data Sharing Statement

Puéchal X, Iudici M, Perrodeau E, et al; French Vasculitis Study Group. Rituximab vs cyclophosphamide induction therapy for patients with granulomatosis with polyangiitis. *JAMA Netw Open*. 2022;5(11):e2243799. doi:10.1001/jamanetworkopen.2022.43799

Data
Data available: No

Additional Information
Explanation for why data not available: Available on request
